# Comparison of two Intranasal Sedatives, Midazolam versus Dexmedetomidine, in Children with High Dental Fear: a Randomized Clinical Trial

**DOI:** 10.30476/DENTJODS.2021.89323.1406

**Published:** 2022-06

**Authors:** Katayoun Salem, Hossein Khoshrang, Elham Esmaeeli, Mona Vatankhah

**Affiliations:** 1 Dept. of Pediatric Dentistry, Faculty of Dentistry, Tehran Medical Sciences, Islamic Azad University, Tehran, Iran; 2 Dept. of Pediatric Dentistry, Guilan Dental school, Guilan University of Medical Sciences, Rasht, Iran; 3 Dept. of Anesthesia, Guilan Medical School, Guilan University of Medical Sciences, Rasht, Iran; 4 Dentist, Guilan Dental School, Guilan University of Medical Sciences, Rasht, Iran; 5 Pediatric Dentist, Dept. of Pediatric Dentistry, Faculty of Dentistry, Tehran Medical Sciences, Islamic Azad University Tehran, Iran

**Keywords:** Dental fear, Dexmedetomidine, IN sedation, Midazolam

## Abstract

**Statement of the Problem:**

Pharmacologic management of uncooperative behavior is a growing trend in dentistry. Determining the most appropriate drug, route of administration, and proper candidate for sedation have been the goal of several investigations.

**Purpose:**

The aim of this study was to compare the sedative effect of intranasal (IN) sedation of midazolam (MDZ) in compare to dexmedetomidine (DEX) while taking into consideration the effect of dental fear, and psychological status on sedation success.

**Materials and Method:**

This double-blind randomized clinical trial included 92 uncooperative dental patients aged 4-6. Study participants were randomly assigned to receive either 0.2mg/kg IN MDZ or 1µg/kg DEX. Sedation was evaluated using the Houpt sedation rating scale. Vital signs were recorded before and during sedation. Prior to sedation, the level of dental fear was determined through children's fear survey schedule-dental subscale (CFSS-DS). Psychological characteristics were screened using the strengths and difficulties questionnaire (SDQ). Data were analyzed using T-test, Mann-Whitney, Chi-square, and repeated-measures analysis of variance (ANOVA).

**Results:**

Overall ratings of sedation and subscales of sleep, crying, and movement were comparable between groups (*p*> 0.05); however, more acceptable behavior (overall scores (4+5+6) was observed in MDZ group compared to DEX group (64% vs. 47.7%) (*p*= 0.007). All participants were found to have abnormal levels of dental fear (CFSS-DS≥38). However, according to SDQ, the study participants have mainly shown normal behavioral status. A significant association was found between dental fear and sedation success (MDZ, *p*= 0.001, DEX, *p*= 0.03), while similar findings were not observed for psychological characteristics (MDZ, *p*= 0.09 and *p*= 0.41; DEX, *p*= 0.71 and *p*= 0.53). Physiological parameters remained within normal limits in both groups.

**Conclusion:**

Sedation with IN MDZ resulted in overall behaviors, which were more satisfactory in highly fearful pediatric dental patients. Despite baseline uncooperative behaviors, the psychological status of study participants were close to average and were not associated with sedation failure.

## Introduction

Reduction of fear and behavioral problems during dental procedures by pharmacological techniques is one of the paramount issues in pediatric dentistry [ 1 ]. Currently, midazolam (MDZ) is the prevailing choice of dental sedation. Considerable research attention has been devoted to intranasal (IN) route of administration of MDZ prior to painful or fear-evoking procedures, notably dental procedures. High bioavailability, convenience, and rapid onset make IN administration a promising choice for pediatric patients. Still, the main disadvantage of IN MDZ is noxious sensation in nasal mucosa [ 2 ]. MDZ may also induce paradoxical behavioral reactions in some patients [ 3 - 4 ]. Within this area of investigation, a number of studies have focused on a more recent sedative agent, dexmedetomidine (DEX), an alpha-2 adrenoreceptor agonist, that induces a sedative state known as cooperative sedation mode. During cooperative sedation mode, the patient is aroused after gentle stimulation and with little effort and [ 1 ] returns to sedative state following the removal of stimulus [ 5 - 6 ]. The main advantages of DEX are preservation of respiratory function during sedation, and less nasal irritation through IN route [ 2 , 7 - 8 ]. Despite several breakthroughs in pediatric sedation, two main questions remain unanswered: what is the best sedative for in-office dental patients, and who stands to benefit most from a particular sedative drug? 

Most scholars seem to agree that both MDZ and DEX are suitable premedication before general anesthesia and for diagnostic purposes. However, there continues to be debate about their effectiveness in dental treatment for children [ 5 , 9 ]. Within this area of investigation, the role of child emotions such as fear and psychological problems on sedation failure deserves more research attention. It has been shown that MDZ is less effective in highly emotional inflexible or psychosomatically problematic children [ 9 - 11 ], however; the role of such variables on DEX sedation is not well understood. 

The aim of this study was twofold. Our first goal was to evaluate the safety and efficacy of IN MDZ and DEX in outpatient dental treatments. The second endpoint was to determine the role of dental fear, and behavioral problems on sedation success through valid and practical tools.

## Materials and Method

### Study design

This double blind randomized controlled clinical trial was conducted according to declaration of Helsinki from August 2018 to June 2019, (ethical approval number#3920422813). All patients consented to participate to the study. Signed consent forms were obtained from participants’ parents or their guardians. The double-blind randomized parallel group clinical trial was registered prior to patient enrollment.

### Patients and setting

Participants in this study were selected based on the history of definitely uncooperative behavior during a last dental visit. Ninety-two normal healthy children (ASA I) aged 4-6 that required at least one treatment session with local anesthesia, were randomly assigned to one of two study groups (MDZ=50, DEX=42) using block randomization. 

### Patients’ allocation

**Figure 1:**
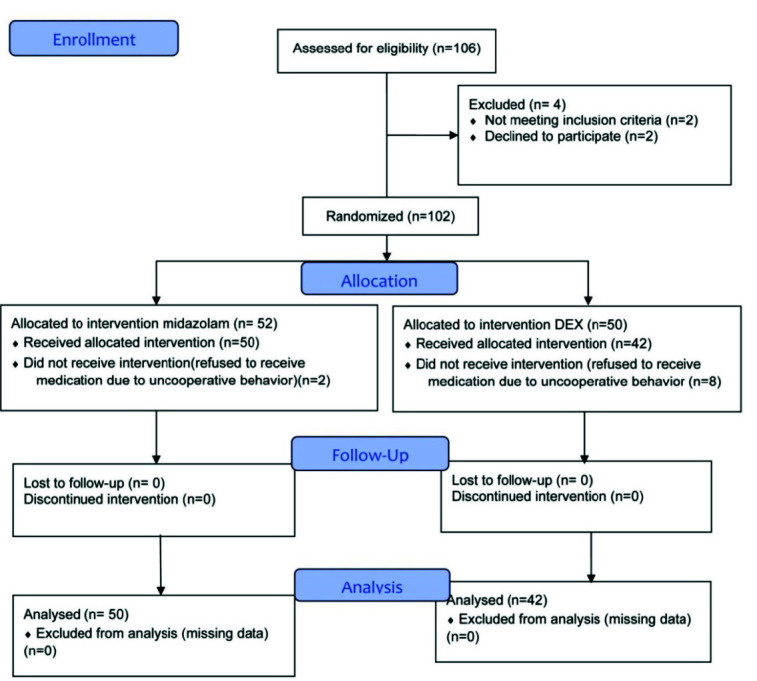
CONSORT Flow Diagram

The CONSORT flow diagram of patient allocation is presented in Figure 1. Sample size was calculated according to Ghali *et al*. [ 12 ]. Sample size of 42 per group provided a 90% power at 0.05 level of significance. Exclusion criteria were allergy to sedative medications in the child or family, and mental disabilities.

### Intervention

### Dental fear and psychological problems assessment

Prior to sedation, parents completed two questionnaires in which they provided information about child dental fear and psychological problems. Dental fear was assessed through the Persian version of children's fear survey schedule-dental subscale (CFSS-DS). The psychological status was screened by the strengths and difficulties questionnaire (SDQ) [ 13 - 14 ]. CFSS-DS has 15 five-choice questions; 1 indicates “no fear at all” while 5 indicates “highly fearful”. Scores ranges from 15 to 75 and scores of 38 or higher indicate abnormal levels of fear.

The SDQ is a screening tool for psychological and behavioral problems. The questionnaire divides the behavior into two categories of child strengths and child difficulties. SDQ has 25 items and each can be answered as “not true”, “relatively true”, and “completely true”. Higher total or subscale scores (except the pro-social domain) indicate higher risk of problems while lower scores in each subscale did not eliminate the possibility of problems. The questionnaire divides behavior into four categories of difficulties; emotional symptoms, conduct problems, hyperactivity and peer problem. SDQ also appraises the strengths of the child by evaluating the pro-social behavior [ 14 ]. 

Sedation assessment was conducted using Houpt behavioral rating scale. This scale divides behavior to subgroups of sleep, crying, movement, and overall behavior [ 15 ]. The scale is presented in detail in Table 1.

**Table 1:** Sedation scores of patients in two groups

Variable / Time		MDZ (n=50)	DEX (n=42)	P^u^
Sleep	1.Fully awake, alert	32(64.0)	25(59.5)	0.65
	2.Drowsy, disorientated	16(32.0)	15(35.7)	
	3.Asleep	2(4.0)	2(4.8)	
Movement	1.Violent movement, interrupting treatment	3(6.0)	2(4.8)	0.87
	2.continuous movement, making treatment difficult	8(16.0)	8(19.0)	
	3.controllable movement that does not interfere with treatment	18(36.0)	15(35.7)	
	4.No movement	21(42.0)	17(40.5)	
Crying	1.Hysterical crying that demands attention	7(14.0)	8(19.0)	0.56
	2.Continuous, persistent crying that makes treatment difficult	15(30.0)	13(31.0)	
	3.Intermittent, mild crying that does not interfere with treatment	14(28.0)	10(23.8)	
	4.No crying	14(28.0)	11(26.2)	
Overall Behavior	1.Aborted, no treatment rendered	1(2.0)	2(4.8)	0.98
	2.Poor, treatment interrupted; only partial treatment completed	5(10.0)	2(4.8)	
	3.Fair, treatment interrupted; but eventually all completed	12(24.0)	18(42.9)	
	4.Good, difficult, but all treatment performed	22(44.0)	4(9.5)	
	5.Very good, some limited crying or movement	6(12.0)	12(28.6)	
	6.Excellent, no crying or movement	4(8.0)	4(9.5)	
Acceptable overall behavior	Scores 4+5+ 6	32(64)	20(47.7)	0.005**
Unacceptable overall behavior	Scores 1+2+ 3	18(36)	22(52.3)	

**) significant level in 0.01.

*) significant level in 0.05.

u ) Mann-Whitney U test.

### Sedation regimen

Group DEX (*n*=42) received 1 mcg/kg of IN DEX (Precedex® Hospira, Inc, Lake Forest USA, maximum dose 25 mcg, equal to 0.25ml) and group MDZ (*n*=50) received 0.2 mg/kg of IN MDZ (MIDAMAX 5mg/1ml AMP tehranchemie.co. Iran, max dose 5mg, equal to 1ml), using a mucosal atomizer. The drugs were prepared by adding 0.9% normal saline to a final diluted volume of 1 ml and were then sprayed to each nostril in 10-second-intervals by a trained nurse under the supervision of an anesthesiologist (H.KH). The nurse, observer (E.S) and practitioner (K.S) were blind to the administered drug. Patients remained in a pre-operative holding area accompanied by a parent and under the supervision of a nurse for 15 and 45 minutes for MDZ and DEX, respectively. Subsequently, patients were moved to the dental unit. All dental treatments were performed by a pediatric dentist (K.S). Pulp treatment was performed using local anesthesia (lidocaine 2%, epinephrine 1/80000; Xylopen®.Iran). Sedation was assessed from the beginning of treatment using the Houpt scale by an independent observer (E.S) and the lowest score on each item was recorded. The details of Houpt scale are presented in Table 1.

### Physiological parameters

Saturation rate of peripheral blood oxygen (SpO2), heart rate (HR), respiratory rate (RR), systolic (SBP) and diastolic blood pressure (DBP), were measured prior to sedation and every 15 minutes during treatment. 

### Data analysis

Kolmogorov–Smirnov test with Lilliefors correction was used to determine the normality of distribution. The normally distributed data were compared using t-test, and in case of skewed distribution, Mann-Whitney U test was used. Qualitative data was analyzed using Chi-square test. Linear models with repeated measures were chosen for overall assessment of physiological parameters. The Cronbach's alpha for intra-observer agreement for sedation assessment was 93%. The software of choice in this study was SPSS v.18 and the significance level of the tests was less than 5% (results less than 5 and 1% were marked with a "*" and a "**" sign, respectively). 

## Results

### Characteristics of participants

A total of 92 patients participated in this study. Basic demographic data of participants is presented in Table 2. 

**Table 2:** Basic demographic data of study participants

Variable		MDZ (n=50)	DEX (n=42)	*p*
Gender	Male	22(44.0)	16(38.1)	0.56^p^
	Female	28(56.0)	26(61.9)	
Age (Y)		5.4±1.3	5.3±0.9	0.71^t^
Weight (kg)		18.4±3.2	19.5±3.7	0.14^t^
Drug acceptance	Good	20(40.0)	38(90.5)	0.0001u**
	Fair	19(38.0)	4(9.5)	
	Poor	11(22.0)	0(0.0)	

**) significant level in 0.01.

*) significant level in 0.05.

t ) Independent Sample t test.

u ) Mann-Whitney U test.

p ) Pearson Chi-Square

### Main outcomes

### Physiological parameters

Table 3 presents the physiological parameters. Among them HR significantly decreased in both groups. Bonferroni correction confirmed HR changes in both groups.

**Table 3:** Mean values of physiological parameters

Variable / Time		MDZ (n=50)	Dexmedetomidine (n=42)	Normal (3-6 yr)	P^t^	PRRB
SPO2	Pre	97.6±1.6	96.8±2.0		0.023*	0.0001**
	0	97.8±1.3	96.8±1.7		0.001**	
	15	97.7±1.4	96.9±1.9	95>	0.015*	
	30	97.8±1.5	96.5±1.7		0.0001**	
	*p* Value (PRRW)	0.84	0.71			
HR	Pre	108.5±14.8	103.7±15.1		0.127	0.06
	0	111.6±15.3	106.5±14.9		0.113	
	15	106.9±13.0	106.2±13.0	65-110	0.813	
	30	106.5±15.4	99.3±12.3		0.016*	
	*p* Value (PRRW)	0.018*,2&4	0.001**,2&4,3&4			
RR	Pre	24.6±3.6	24.5±3.6		0.870	0.98
	0	24.8±3.6	25.0±2.7		0.813	
	15	23.9±3.1	24.4±3.2	20-25	0.387	
	30	24.3±3.9	23.6±2.9		0.386	
	*p* Value (PRRW)	0.19	0.052			
Systolic (mg)	Pre	107.9±17.3	113.6±15.2		0.098	0.21
	0	110.9±12.3	111.7±14.4	95-110	0.767	
	15	105.9±14.3	109.1±16.9		0.319	
	30	108.0±8.8	109.2±12.2		0.569	
	*p* Value (PRRW)	0.15	0.17			
Diastolic (mg)	Pre	65.4±12.6	71.1±14.1		0.050	0.03*
	0	67.8±14.4	70.0±16.1	60-75	0.497	
	15	63.7±11.5	67.5±14.9		0.179	
	30	63.4±11.5	68.8±10.5		0.021*	
	*p* Value (PRRW)	0.06	0.58			

SPO2 (Peripheral oxygen saturation), HR (Heart rate), RR (Respiratory rate)

**) significant level in 0.01.

*) significant level in 0.05.

t ) Independent Sample t-test.

RRW ) Repeated Measurement analysis of Variance (Tests of Within-Subjects Effects).

RRB ) Repeated Measurement analysis of Variance (Tests of Between-Subjects Effects).

24 ) Compare between time 0 and time 30.

34 ) Compare between time 15 and time 30.

Adapted from Dentistry for child and adolescent, 10 ed, Philadelphia, 2016, Elsevier Science.

### Sedation scores

As shown in Table 1, the two groups did not show any significant difference in terms of drowsiness (*p*=0.65), movement (*p*= 0.87), crying (*p*= 0.56), and overall behavior (*p*= 0.98). The overall behavior was further divided into categories of unacceptable (scores 1+2+3) and acceptable (scores 4+5+6). The behaviors observed in the MDZ group were more acceptable compared to the DEX group (*p*< 0.005). Acceptance of medication in the DEX group was superior to the MDZ group (90.5% versus 40.0%, *p*< 0.0001).

### CFSS-DS

The mean (sd) values of CFSS-DS are shown in Table 4. A significant association was found between CFSS-DS and unacceptable behavior: MDZ *p*< 0.001, DEX *p*=0.03. 

**Table 4:** Mean (SD) values of dental fear (CFSS-DS), and strength and difficulties status questionnaires

Variable		MDZ (n=50)	DEX (n=42)	Normative scores	P^u^
CFSSDS		39.4±8.7	44.1±9.6	38<	0.03*
SDQ1	Emotional Symptoms	2.3±1.8	2.1±1.5	CA 0-3	0.95
				SR/L 4	
				H/L 5-6	
				VH/VL7-10	
SDQ2	Conduct Problems	3.5±1.3	3.6±1.5	CA 0-2	0.94
				SR/L 3	
				H/L 4-5	
				VH/VL6-10	
SDQ3	Hyperactivity	4.2±1.7	3.8±1.8	CA 0-5	0.26
				SR/L 6-7	
				H/L 8	
				VH/VL 9-10	
SDQ4	Peer Problem	1.6±1.4	1.6±1.5	CA 0-2	0.65
				SR/L 3	
				H/L 4	
				VH/VL 5-10	
SDQ5 Strength	Pro-social Behavior	6.8±1.5	6.7±1.4	CA 8-10	0.83
				SR/L 7	
				H/L 6	
				VH/VL 0-15	
Total difficulties	Difficulties sum (1-4)	11.7±3.9	11.1±4.3	CA 0-13	0.47
				SR/L 14-16	
				H/L 17-19	
				VH/VL 20-40	

CA=close to average, SR/L: slightly raised or lowered, H/L: high/low. VH, VL: very high/very low

### SDQ

The results obtained from study participants and the normative SDQ values are summarized in Table 4. No statistically significant difference was observed regarding SDQ scores. The SDQ domains were close to average, with the exception of slightly released conduct problems and high pro-social behavior scores. There was no significant association between overall behavior and total difficulties, or strengths (pro-social behavior) in either group (MDZ: *p*= 0.41 and *p*= 0.09; DEX: *p*= 0.53 and *p*= 0.71).

## Discussion

The general picture emerging from the analysis is that IN administration of 0.2mg/kg MDZ and 1mcg/kg DEX have similar sedative effects in pediatric dental sedation. The comparable ratings of sedation gained by the two groups were in coordination with other studies [ 14 , 16 - 18 ]. However, an interesting finding was that when the overall behavior was divided into acceptable (good, very good and excellent) and unacceptable (aborted, poor and fair), MDZ was superior to DEX (64% vs. 47.7 %). Our findings are consistent with other studies showing MDZ was associated with less combative behavior than DEX when used as a procedural sedation [ 3 , 8 ]. 

While premedication with 1 mcg/kg DEX has led to satisfactory results in parental separation, mask induction, and intravenous cannulation before general anesthesia, using similar doses in dental pediatric procedures has not yielded the same results [ 3 , 16 - 18 ]. A possible explanation for this discrepancy might be the painful and irritating nature of dental procedures. A higher dose of 2mcg/kg is advocated for procedures that are associated with stress and pain [ 19 ]. This finding may further be interpreted in light of pharmacodynamics and clinical behavior of patients. MDZ exerts its sedative and anxiolytic effects by releasing the inhibitory neurotransmitter gamma amino butyric acid (GABA) through the benzodiazepine 1 and 2 receptors located in the cerebral cortex and the limbic system. The activation of GABA receptors is regulated by a combination of pharmacological effects, genetics and clinical behavior [ 15 - 16 ], whereas the site of action of DEX is in locus coeruleus. Locus coeruleus is an area of brain that plays an important role in formation and retrieval of fear evoking memories [ 17 ]. Hence, a related idea, which might explain this discrepancy, is the presence of dental fear. Dental fear is a normal emotional reaction to threatening stimuli in the dental situation. It is noteworthy that fear can have deterimental effects on mild and moderate sedation [ 3 , 7 - 8 , 13 ]. 

In the present study, both groups experienced abnormal levels of dental fear; however, by chance the baseline score of CFSS-DS was higher in DEX group. Synergistic effects of higher baseline fear and procedural stimulants of fear, such as injection, may justify the more unacceptable behaviors in DEX group [ 14 ]. 

In addition to dental fear, the psychological status of the child may play a role in the failure of sedation [ 11 , 16 ]. The findings from the SDQ show particularly interesting patterns. Contrary to our expectations, the results showed no significant psychological problems and no association with overall sedation ratings among study population. Only the SDQ’s subscale of conduct problems was slightly raised. Conduct problems encompass behavioral features such as temper tantrums; however, these children are generally obedient, and usually do what adults request [ 14 ]. Hence, it would be suggested that this characteristic might be helpful when a combination of pharmacological and non-pharmacological behavior management techniques is used. Furthermore, the SDQ results demonstrated high scores of pro-social behaviors. Pro-social behaviors demonstrated the strength points of child by disclosing the characteristics such as helpfulness and consideration of others’ feelings. The high score of pro-social behaviors was an additional factor that supports this conclusion [ 14 ]. Some studies reported more failure of sedation with MDZ among children who are highly emotional, inflexible, or highly impulsive [ 9 - 11 ]. Since DEX is a relatively new sedative in the field of dentistry, there are no similar studies on its effectiveness regarding child behavioral characteristics [ 14 - 16 ].

As a result, in the absence of psychological problems, the only contributing factor in failure of sedation in the present study was dental fear. To summarize, MDZ was superior to DEX in successful management of fearful pediatric dental patients. Another concern was the level of drowsiness. Although DEX induces a sedative state resembling to early stages of non-rapid eye movement sleep that patient can be easily awakened but returns to sedative state by removing the stimulus. MDZ is a sleep promoting agent; only two patients in either group were observed fully asleep. The predominance of non-drowsy state could be associated with the arousing nature of dental treatment. Moreover, it is not surprising to find patients with only limited signs of sleepiness since low doses of drugs were used [ 15 ]. 

Concerning patients' movements, both sedatives controlled the disturbing movement properly. From the domains of Houpt sedation rating scale, crying remained the main obstacle in successful sedation for both drugs. With respect to safety, no case of hypoxemia, hypotension, or bradycardia was observed, in concordance with previous studies [ 3 , 20 - 21 ]. The main hemodynamic finding was an increase in HR at the beginning of the treatment followed by a slow slope decrease. The decline was more pronounced in DEX and was assumed a physiologic response to this agent due to decrease in epinephrine and norepinephrine levels [ 20 ]. DEX encompasses a more sympathetic tone-decreasing capacity than MDZ [ 21 - 23 ]. Remarkably, HR demonstrates the greatest variability among the physiological parameters and within subjects. HR is more dependent on physiological demands and treatment procedures in children. Notably, increased HR is the most observed autonomic sign of dental fear [ 20 , 24 - 26 ]. 

The lower acceptance rate of MDZ in comparison to DEX is one of its main drawbacks. Even when diluted with 0.9% saline, MDZ was associated with unpleasant sensation in nasal mucosa. Our findings are consistent with previous studies showing the stinging sensation of MDZ [ 3 - 4 , 27 ]. One of the limitations of the present study was the predominance of close to average ratings of SDQ. Hence, the role of mental health problems in sedation outcomes should be studied more extensively. The sample size of the present study was calculated according to Ghali *et al*. [ 12 ], based on sedative variables. Recalculation of study power determined that using the current sample size achieves a 90% power to detect the effectiveness of the two sedatives on overall behavior, with an effect size of 0.42 using 5 degrees of freedom (Chi-Square, *p*< 0.05). However, due to lack of association between SDQ and sedation failure, the findings of this study regarding psychological problems and failure of sedation should not be generalized beyond the study population until further studies.

## Conclusion

Our data provide compelling evidence that IN sedation with MDZ results in more acceptable overall behavior than DEX in pediatric patients with high dental fear. Among the ingredients of sedation failure, crying was playing a key role. The effect of psychological characteristics on sedation success needs more investigation.

## Conflict of Interest

None declared.
